# Networking of predicted post-translational modification (PTM) sites in human EGFR

**DOI:** 10.6026/97320630015448

**Published:** 2019-07-31

**Authors:** Arshi Malik, Sarah Afaq, Afaf S Alwabli, Khalid Al-ghmady

**Affiliations:** 1 Department of Clinical Biochemistry, College of Medicine, King Khalid University, Abha Kingdom of Saudi Arabia-61421; 2 Department of Biological Sciences, Faculty of Science, King Abdulaziz University, Jeddah 21589, Saudi Arabia

**Keywords:** Cancer, EGFR, PTMs, pathways, interaction

## Abstract

Epidermal growth factor receptor (EGFR) binds to EGF activating tyrosine phosphorylation through receptor dimerization prompting uncontrolled multiplication. Domain organization, secondary structure combinations in motifs and interactome define such transitory changes responsible for the multi-functionality of human EGFR. We report the predicted phosphorylation sites on Ser, Thr and Tyr residues in addition to 74 auto-phosphorylation sites on Tyr in human EGFR. These data suggest a complex interplay between phosphorylation types for modification resulting in the modulation of human EGFR functionality. It is of further interest in future to thoroughly understand the associated data to clarify the various roles played by post translational modifications (PTM) in human EGFR.

## Background

The EGFR linked to cancerous growth was perceived when the changing v-ErbB oncogene of the avian erythroblatosis infection was observed to be a homolog of human EGFR [1,2]. The v-erbB oncogene was found to contain recombination of the transmembrane and cytoplasmic spaces of the EGFR [3], connecting EGFR distortions to cancerous growth. Notwithstanding transformations, over expression of EGFR was then seen to advance disease movement, first in carcinomas [4,5], and later on in sarcomas, non-little cell lung malignancy (NSCLC) [6] and dangerous gliomas [7]. Various human diseases are not yet linked to unique qualities; however, they emerge from complex collaborations among different hereditary variations [8]. Consequently, to understand infection, a system of key players that are identified with the infection, their communications, for instance, through natural pathways, must be considered. It is of interest to study the EGFR flagging pathway and EGFR PPIs in an RCC population [9,10]. The EGFR is a receptor tyrosine kinase that manages the central procedures of cell development and separation [11]. A similarly significant and dynamic PTM adjustment is distinguishable in almost all higher eukaryotic living beings [12]. It is a pervasive alteration that is controlled by O-GlcNAc transferase (OGT) (adds O-GlcNAc to protein spine) and O-GlcNAcase. The complex interplay among phosphorylation and O-GlcNAc adjustment on the equivalent or neighboring deposits, the yin-yang destinations, has been seen in a few atomic and cytoplasmic proteins [13], and proof of O-GlcNAc alteration in EGFR type III has been tentatively checked [14]. This elective alteration of Ser/Thr buildups by O-GlcNAc and phosphate frequently results in practical switches of a protein [14]. In this study, we have distinguished conserved domain, secondary structure combinations in motifs, interacting partners, and posttranslational modifications, including phosphorylation sites and other parameters in human EGFR. Interplay between phosphorylation modification on Ser and Thr residues in the EGFR occurs, suggesting an essential role in the functional regulation of human EGFR.

## Methodology

### Human EGFR domain and secondary structure prediction:

The amino acid sequence of EGFR was retrieved from the genome database at NCBI. Domain organization was assigned using SMART, Pfam, Prosite, InterProScan, and PANTHER programs [15-17]. The secondary structure perdition of human EGFR protein (containing several helices, strands, and coils) and membrane orientation was completed using MEMEMBED [18].

### Functional protein association network using STRING:

The amino acid sequence of EGFR protein (Homo sapience) was submitted to the STRING DB version 10. The protein-protein interaction study was done using high sureness parameters (0.9) and filtering number of interfacing assistants to twenty. Different sources have been used to predict these associations, for instance, central databases give known interchanges, and physically curated databases provide pathway learning. Known data are benchmarked and adjusted against past data, using the strange state groupings given by the manually curated Kyoto Encyclopedia of Genes and Genomes (KEGG) pathway maps [19,20].

### Analysis of human EGFR metabolic pathways:

Human EGFR and its downstream signaling proteins are analyzed using the Simple Modular Architecture Research Tool (SMART) (https://smart.embl.de/) to identify different metabolic pathways. SMART software (https://smart.embl.de/) was used to analyze information about predicted molecular pathways with biological functions.

### Analysis of human EGFR metabolic pathways:

Posttranslational modifications analysis (PTM) in human EGFR: PTMs regularly influence the capacity of an adjusted protein and in this way expand the learning on potential PTMs of a protein for the understanding of their participation [21].The retrieved sequence of EGFR protein is submitted to the Simple Modular Architecture Research Tool (SMART) (https://smart.embl.de/). There are 127 PTMs in human EGFR. The sequence data, used for prediction of phosphorylation and modification sites in human EGFR, were retrieved from the unipot sequence database. NetphosK 1.0 (http://www.cbs.dtu.dk/ services/NetPhosK/) was used to predict the capability of kinases destined to phosphorylate Ser, Thr, and Tyr in the human EGFR [21].

## Results and Discussions:

### Domain arrangements and PSIPred secondary structure analysis in human EGFR:

**Figure 1:**
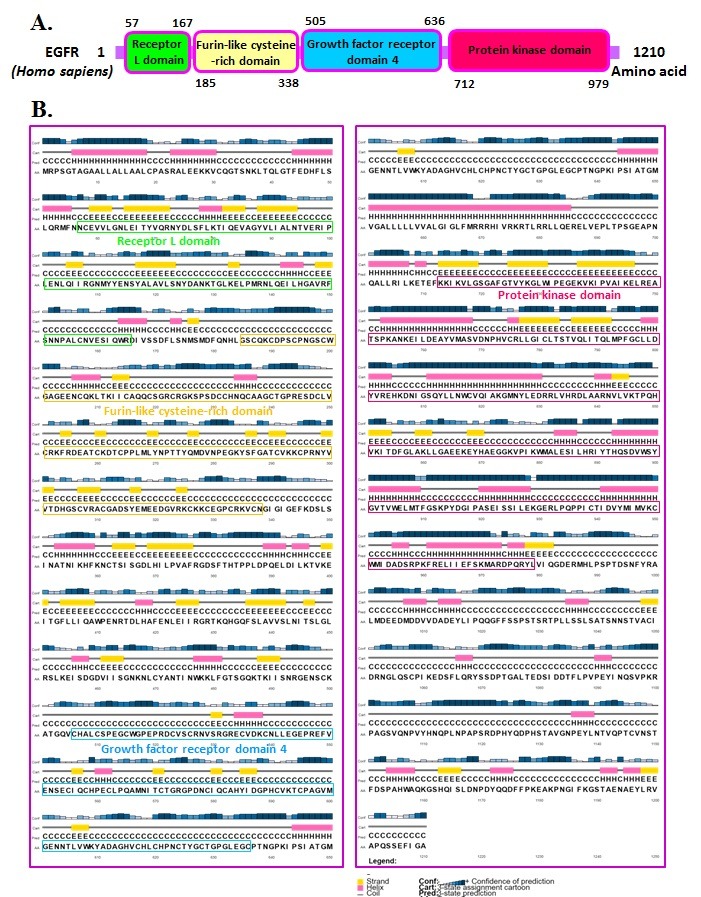
Domain organization in human EGFR is shown. (A) Each conserved domain is given in boxes with different colors. The numbers refers to the amino acids separating the various domains. (B) Secondary structure prediction using the PSIPRD server (http://bioinf.cs.ucl.ac.uk/psipred/) is shown. The graph represents the secondary structures in human EGFR.

We completed a study of the amino acid (1210) arrangement of domains in human EGFR protein. EGFR protein contains four domains that are receptor L domain, a furin-like cysteine-rich domain, growth factor receptor domain, and a protein kinase domain as shown in Figure 1A. The secondary structure prediction by PSIPRED demonstrates that presence of strands, α-helix, and loops in the human EGFR (Figure 1B).

### Human EGFR interacting protein network:

**Figure 2:**
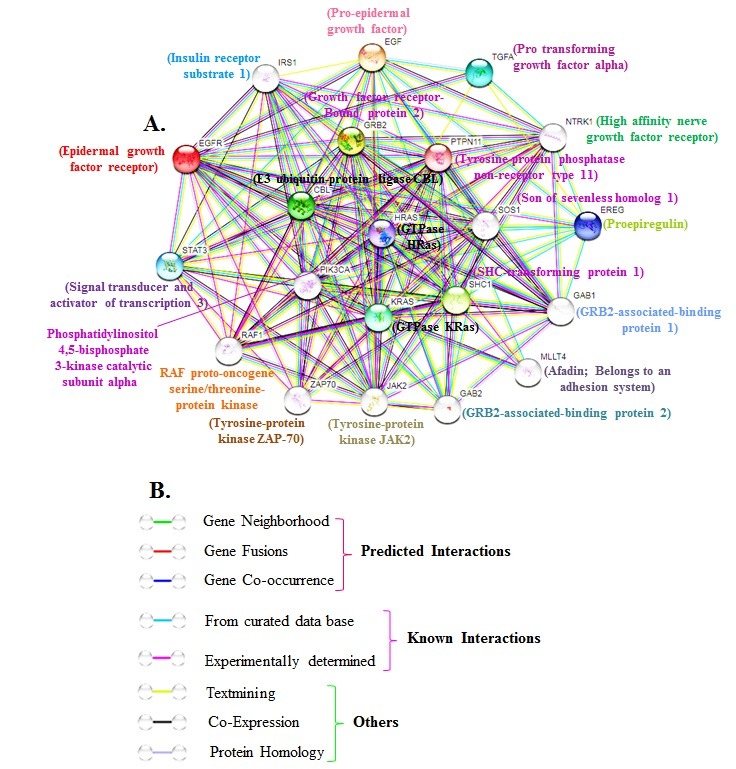
Protein-protein association networks analysis. (A) Interacting protein prediction of EGFR using STRING v10.database was shown. Hued lines between the proteins demonstrate the different kinds of association. Protein nodes with known structure data are shown. (B) Explanations for interactions are given.

The EGFR protein network obtained from the STRING database consists of proteins like EGF, GRB2, SHC1, cas-Br-M (murine) ecotropic retroviral transforming sequence (CBL), transforming growth factor alpha (TGFA), protein tyrosine phosphatase, nonreceptor type 1 (PTPN1), signal transducer and activator of transcription 3 (acute-phase response factor) (STAT3), ubiquitin C (UBC), v-erb-b2 erythroblastic leukemia viral oncogene homolog 2 (ERBB2), phospholipase C, gamma 1 (PLCG1), ERBB receptor feedback inhibitor 1 (ERRFI1), Epidermal growth factor receptor pathway substrate 15 (EPS15), protein tyrosine phosphatase and several non-receptor types. The STRING version 10 server with the parameters mentioned in methods was utilized (http://stringdb. org/) in this study [22-24]. Thus, the network analysis identified twenty interacting proteins. The results show that EGFR protein interacts with signal transducer and activator of transcription 3, phosphatidyl-inositol 4,5-bisphosphate 3-kinase catalytic subunit alpha, RAF proto-oncogene serine/threonine-protein kinase, Tyrosine-protein kinase ZAP-70, Tyrosine-protein kinase JAK2, GRB2-associated-binding protein 2, GRB2-associated-binding protein 1, Afadin; Belongs to an adhesion system, proepiregulin, High affinity nerve growth factor receptor, Pro transforming growth factor alpha, Pro-epidermal growth factor, Insulin receptor substrate 1, growth factor receptor-bound protein 2, Tyrosineprotein phosphatase non-receptor type 11, Son of sevenless homolog 1, SHC-transforming protein 1, GTPase KRas, GTPase HRas and E3 ubiquitin-protein ligase CBL (Figure 2A).

### Analysis of metabolic pathway:

**Figure 3:**
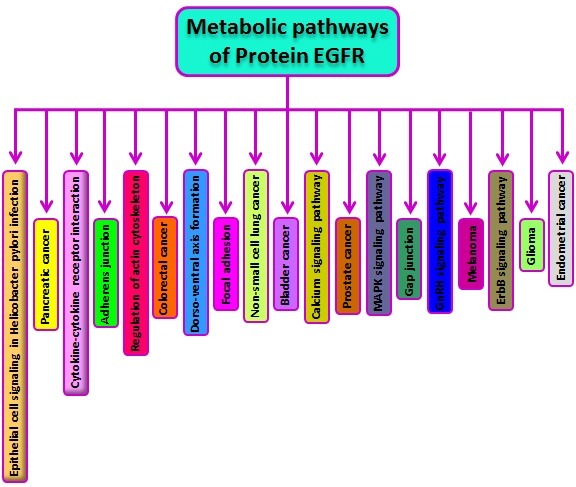
The human EGFR involved in different metabolic pathways is shown. The pathway data illustrated here was taken from different sources to provide a comprehensive overview.

Human EGFR and receptor tyrosine kinase binding ligands of the EGF family help in activating several signaling cascades to convert extracellular signals into appropriate cellular responses. Known ligands include EGF, TGFA/TGF-alpha, amphiregulin, epigen/EPGN, BTC/betacellulin, epiregulin/EREG, and HBEGF/ heparin-binding EGF. The EGFR protein involved in many different pathways is shown. The graphical representation shows the details of pathways with components having annotations such as non-small cell lung cancer, melanoma, calcium signaling pathway, gap junction, regulation of actin cytoskeleton, glioma, ErbB signaling pathway, prostate cancer, dorso-ventral axis formation, focal adhesion, pancreatic cancer, GnRH signaling pathway, MAPK signaling pathway, endometrial cancer, cytokinecytokine receptor interaction, colorectal cancer, epithelial cell signaling in Helicobacter pylori infection, adherens junction and bladder cancer (Figure 3). There are three essential pathways which are playing a vital role that is non-small cell lung cancer pathway having lung cancerous growth as a leading source of disease. Nonlittle cell lung disease (NSCLC) represents roughly 85% of lung malignancy and speaks to a heterogeneous gathering of tumors, comprising for the most of squamous cell (SCC), adeno (AC) and enormous cell carcinoma. Sub-atomic instruments adjusted in NSCLC incorporate initiation of oncogenes, for example, K-RAS, EGFR and EML4-ALK, and inactivation of tumor suppressor qualities, for example, p53, p16INK4a, RAR-beta, and RASSF1 [22,23]. EGFR protein has a crucial role in the calcium signaling pathway, Ca2+ that enters the cell from the outside is an essential wellspring of sign Ca2+. Section of Ca2+ is driven by the nearness of an enormous electrochemical inclination over the plasma film. Cells utilize this outside wellspring of sign Ca2+ by initiating different section channels with generally various properties. The voltageworked channels (VOCs) are found in volatile cells and produce the quick Ca2+ transitions that control quick cell forms. There are numerous other Ca2+-section channels, for example, the receptorworked channels (ROCs), for instance [22]. The MAPK signaling pathway, the mitogen-actuated protein kinase (MAPK) course is an exceptionally preserved module that is engaged with different cell capacities, including cell expansion, separation, and relocation. Vertebrates express in any event four mainly managed gatherings of MAPKs, extracellular sign related kinases (ERK)- 1/2, Jun amino-terminal kinases (JNK1/2/3), p38 proteins (p38alpha/beta/gamma/delta) and ERK5, that are initiated by explicit MAPKKs: MEK1/2 for ERK1/2, MKK3/6 for the p38, MKK4/7 (JNKK1/2) for the JNKs, and MEK5 for ERK5. Each MAPKK, in any case, can be initiated by more than one MAPKKK, expanding the intricacy and assorted variety of MAPK flagging. Each MAPKKK gives responsiveness to unmistakable boosts. For instance, actuation of ERK1/2 by development factors relies upon the MAPKKK c-Raf. However, different MAPKKKs may initiate ERK1/2 in light of genius fiery upgrades. The NMDA (N-methyl- D-aspartate) receptors (NMDARs) that react to glutamate is seen. There are additionally second-courier worked channels (SMOCs) and store-worked channels (SOCs) in the network [24-26].

### Posttranslational modification in human EGFR:

**Figure 4:**
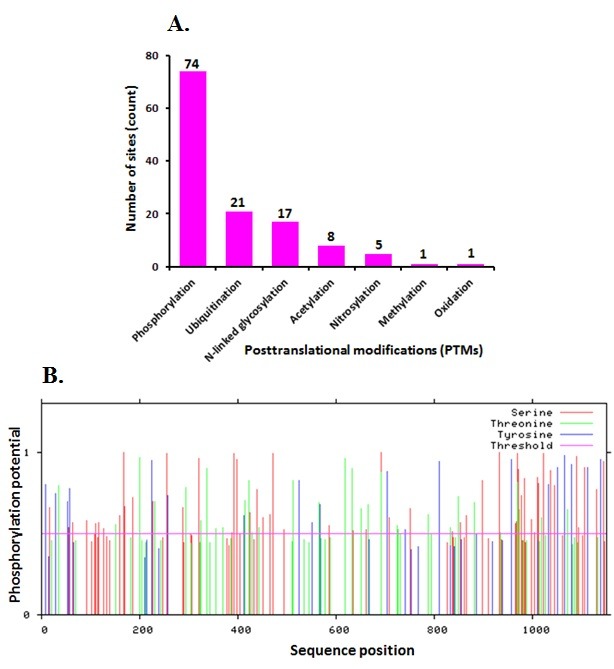
Posttranslational modifications (PTM) human EGFR is shown. (A) Predicted functional associations between protein posttranslational modifications (PTMs) are shown. Each number in the graph indicates the PTMs site count. (B) Graphic presentation of the potential phosphate alteration in human EGFR is shown. The red vertical lines are the phosphorylated Ser residues; the green lines show the phosphorylated Thr residues; the blue line illustrates the phosphorylated Tyr residues. The pink flat line is the threshold for alteration potential.

PTMs are a resource of known and predicted functional associations between protein post-translational modifications (PTMs) within and between interacting proteins [27]. It currently contains seven PTMs that are phosphorylation (74 counts), ubiquitination (21 counts), N-linked glycosylation (17 counts), acetylation (8 counts), nitrosylation (5 counts), methylation (1 count) and oxidation (1 count). The total numbers of the count are 121 (Figure 4A). The graph gives the types of PTMs and their count. Prediction results of human EGFR by Netphos 2.0 for Ser, Thr, and Tyr are given in Figure 4B. These data show a high phosphorylation potential in human EGFR and minimum in methylation and oxidation potentials. The data from NetphosK 1.0 for foreseeing the capability of kinases associated with the EGFR's auto-phosphorylation locales demonstrate that EGFR is having the central potential for kinase functioning in the network.

## Conclusion

Human EGFR and receptor tyrosine kinase binding ligands of the EGF family are involved in several signaling cascades to convert extracellular signals into appropriate cellular responses. Known ligands include EGF, TGFA/TGF-alpha, amphiregulin, epigen/EPGN, BTC/betacellulin, epiregulin/EREG, and HBEGF/ heparin-binding EGF. The phosphorylated receptor recruits adapter proteins, which in turn activates downstream signaling cascades. We have shown the significance of PKC and Ca++ and their interchange in the differential control of human EGFR's flagging in this study. We have also estimated the number locales in the human EGFR. It is of further interest to thoroughly understand the associated data to clarify the various roles played by PTM in human EGFR.
